# Sensing and Integration of Erk and PI3K Signals by Myc

**DOI:** 10.1371/journal.pcbi.1000013

**Published:** 2008-02-29

**Authors:** Tae Lee, Guang Yao, Joseph Nevins, Lingchong You

**Affiliations:** 1 Department of Biomedical Engineering, Duke University, Durham, North Carolina, United States of America; 2 Institute for Genome Sciences and Policy, Duke University, Durham, North Carolina, United States of America; 3 Department of Molecular Genetics and Microbiology, Duke University Medical Center, Durham, North Carolina, United States of America; Virginia Polytechnic Institute, United States of America

## Abstract

The transcription factor Myc plays a central role in regulating cell-fate decisions, including proliferation, growth, and apoptosis. To maintain a normal cell physiology, it is critical that the control of Myc dynamics is precisely orchestrated. Recent studies suggest that such control of Myc can be achieved at the post-translational level via protein stability modulation. Myc is regulated by two Ras effector pathways: the extracellular signal-regulated kinase (Erk) and phosphatidylinositol 3-kinase (PI3K) pathways. To gain quantitative insight into Myc dynamics, we have developed a mathematical model to analyze post-translational regulation of Myc via sequential phosphorylation by Erk and PI3K. Our results suggest that Myc integrates Erk and PI3K signals to result in various cellular responses by differential stability control of Myc protein isoforms. Such signal integration confers a flexible dynamic range for the system output, governed by stability change. In addition, signal integration may require saturation of the input signals, leading to sensitive signal integration to the temporal features of the input signals, insensitive response to their amplitudes, and resistance to input fluctuations. We further propose that these characteristics of the protein stability control module in Myc may be commonly utilized in various cell types and classes of proteins.

## Author Summary

The transcription factor Myc plays a critical role in regulating diverse cell-fate decisions, including growth, proliferation, and programmed cell death. Underscoring its importance, Myc expression is often found to be deregulated in cancers. However, the dynamic mechanism by which Myc is controlled by its upstream signaling proteins remains unclear. To address this issue, we analyze a well-defined signaling module for Myc regulation using a kinetic model constrained by experimental data and observations. In this module, Myc acts as an integrator of its upstream signals that differentially regulate its stability. We show that this module can enable highly sensitive Myc response to the temporal features of the input signals, but not to their maximum amplitudes. We further suggest that this module represents a generic post-translational mechanism for signal sensing and integration in diverse signaling networks. Our work offers insight into the “design” of natural biological networks and makes predictions that can guide further experimental studies on Myc regulation. Moreover, it defines a simple signal processing unit that may be useful for engineering synthetic gene circuits to carry out cell-based computation.

## Introduction

The proto-oncogene protein Myc is a transcription factor that regulates numerous signaling pathways involved in cell-fate decisions [1]–[4]. Sufficient accumulation of Myc leads to the activation of Cyclin D and cyclin dependent kinases, which subsequently phosphorylate Rb and release E2F. This results in the initiation of DNA replication and cell cycle entry [5]. Excessive accumulation of Myc, however, induces apoptosis [6],[7] when cells are under stress or deprived of growth factors. Finally, Myc also drives cell growth by activating genes that encode cellular metabolic activities, including translational factors, ribosomal proteins and RNAs [8].

**Figure 1:**
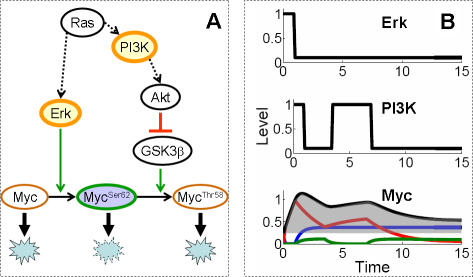
Myc protein stabilization for Myc accumulation. (A) Stimulation with growth factors (GF) leads to activation of Ras and Myc synthesis. Active Ras induces activation of its downstream effector pathways: the MAPK and PI3K pathways. While the synthesized Myc is unstable with short half-life, its stability can be significantly increased via the Ras effector pathways. Active Ras induces Erk that stabilizes Myc by phosphorylation at Ser62. PI3K activation blocks Myc degradation by inhibiting phosphorylation at Thr58 by Gsk3β. As Ras activity declines, Gsk3β initiates phosphorylation of Myc at Thr58 and triggers degradation. Phosphorylation at Thr58 requires prior phosphorylation at Ser62, and phosphorylation at Thr58 induces dephosphorylation at Ser62. (B) Activation patterns of Erk and PI3K determine Myc stability pattern. The three forms of Myc are plotted independently. The unmodified Myc (blue line) and Myc^Thr58^ (green line) accumulate only to a limited level, but stabilized Myc^Ser62^ level increases via phosphorylation (red line). The total Myc level is the sum of the three forms of Myc (black line) and its dynamics are highly correlated with input signals, Erk, and PI3K. We define the shaded area under the Myc curve as “potency”, a measure of Myc accumulation.

Given its importance, Myc activity must be properly controlled in response to different environmental cues. Past studies have suggested that Myc is regulated at multiple levels, including auto-regulation of Myc transcription [9] and post-transcriptional regulation [10],[11]. More recent discoveries indicate that Myc is also dynamically regulated at the protein level by the Ras effector pathways [12]–[15]. These discoveries suggest that Myc protein undergoes a series of modifications that are sequential and irreversible [12]–[14],[16],[17]. More specifically, when Myc is newly synthesized, it is highly unstable and quickly undergoes ubiquitination and degradation [18]. It can be substantially stabilized when phosphorylated at serine 62 (Ser62) by Ras-activated Erk activity (Figure 1A). Subsequent phosphorylation of Myc at threonine 58 (Thr58) by Gsk3β, however, initiates a destabilization process in a sequential manner. This is achieved by a dephosphorylation mechanism by a prolyl isomerase Pin1 and a protein phosphatase PP2A. Once Myc is phosphorylated at Thr58 (Myc^Ser62-Thr58^), Pin1 induces it to undergo conformation changes, which are required for PP2A to dephosphorylate the Ser62 residue (Myc^Thr58^) [14]. To date, this is the only dephosphorylation mechanism identified in the Myc stabilization processes. Destabilization of Myc by Gsk3β can be blocked by the Ras-activated PI3K pathway (Figure 1A).

The unique control of Myc dynamics by sequential phosphorylation allows Myc to integrate upstream signals from Erk and PI3K, which play critical roles in controlling diverse cell fates [14],[19],[20]. Erk often exhibits an early, transient peak of activation upon growth stimulation (Table S1). The peak is followed by varying residual activities, which depend on cell lines and growth factors. This residual Erk is critical in downstream signal encoding. For example, in PC12 cells, a small residual Erk activity, as a result of epidermal growth factor (EGF) stimulation, leads to proliferation. In contrast, a high residual Erk activity as a result of nerve growth factor (NGF) stimulation in the same cell line leads to differentiation [21],[22]. The residual Erk level has also been observed to be critical in regulating c-Fos level in fibroblasts [23].

The PI3K activation pattern depends on cell lines and stimulants, as detailed in Table S2. It is bimodal (having two peaks) in various cell lines including WI38, NIH 3T3, or HepG2 when stimulated by platelet-derived growth factors (PDGF) or fetal bovine serum (FBS) [16],[24],[25]. In contrast, PI3K appears to have only an early, transient single peak in the U-2OS or PVSM cell lines stimulated with other growth stimulants [24],[26]. The bimodal activation of PI3K has been shown to be important for cell cycle regulation [25],[27],[28]. In particular, the second peak has been found sufficient and critical to drive the G1/S transition during cell cycle [16],[27].

The temporal pattern of Myc activation closely correlates with those of Erk and PI3K (Table S3). Myc protein reaches its peak at ∼2 hours after growth stimulation and decreases to and remains at an intermediate value, or hump, for over ∼6 hours before reducing to its basal level [16],[29]. The peak and the hump of Myc coincide with the Erk peak (also the 1^st^ PI3K pulse) and the 2^nd^ PI3K pulse, respectively. These observations suggest that Myc may sense and integrate signals from its two regulators (Erk and PI3K).

To gain insight into this control mechanism, we have constructed a mathematical model to analyze dynamics of Myc accumulation controlled by sequential phosphorylation. Using this model, we aimed to investigate how signaling patterns of Erk and PI3K regulate Myc dynamics at the post-translational level. Also, how robust is Myc dynamics with respect to network parameters, such as phosphorylation and dephosphorylation rate constants? What is unique about this strategy of controlling Myc accumulation by sequentially modulating protein stability? Is this a common strategy by which cells achieve reliable temporal control of key regulatory proteins? By exploring these questions, our work may provide insights into design features of cell signaling networks and guidance for experimental intervention. Conceptually, our model defines a unique module that connects with other models that deal with upstream signaling dynamics leading to the activation of Erk [21] or PI3K [30],[31], as well as downstream dynamics leading to mammalian cell fate decisions [32]–[34] We further propose that post-translation regulation of Myc represents an example of a generic dual-kinase motif. With appropriate parameters, this motif will enable precise temporal sensing of input signals.

## Results/Discussion

### The Base Simulation

The Myc temporal dynamics, simulated with reaction kinetics and base parameter values in Table S4 and S5, was overall consistent with experimental observations in Figure 1B [27],[29]. To achieve this consistency, however, we found that the input signals Erk and PI3K needed to operate at or close to saturation, and there needed to be sufficient residual Erk (Erk_R_) before the second PI3K pulse (See the next section, as well as Tables S4 and S5). In the base simulation (Figure 1B), the total Myc (black line) consisted of unmodified, unstable Myc (blue line), stable Myc^Ser62^ (red line), and unstable Myc^Thr58 ^(green line). Although phosphorylation state affects transactivation capacity [14], the contribution from Myc^Ser62^ to total Myc was much more significant than that from Myc^Thr58^. Therefore, we assumed that the overall transactivation capacity of Myc does not change significantly during Myc modulation. The modification of Myc from its unstable to stable form, then back to unstable form closely followed Erk and PI3K signals. The first peak of Myc coincided with the Erk pulse and the first PI3K pulse. After these initial pulses, unmodified Myc and Myc^Ser62^ recovered to new steady state levels, which depended on the rate constants of Myc synthesis, phosphorylation, and degradation. Before Myc^Ser62^ reached its new steady state, however, PI3K pulse became activated for the second time and prevented Myc^Ser62^ from converting to the unstable form, sustaining total amount of Myc at high level. Once the second PI3K pulse subsided, Myc^Ser62^ was turned to Myc^Thr58^ via phosphorylation by Gsk3β. In other words, while Erk and the first peak of PI3K determine initial Myc accumulation, the second peak of PI3K prevents Myc from receding to a lower level, thus fine-tuning the Myc level.

As Myc accumulation was determined by conversion between its unstable forms and stable form, we expected Myc accumulation to depend on the degradation rate constant of each form. As a quantitative estimate for Myc accumulation, we used Myc potency, the shaded area in Figure 1B. If Myc became stabilized quickly and remained stabilized for an extended period of time, Myc potency would be high. In contrast, slow stabilization and quick destabilization would yield small potency. Consistent with these notions, our sensitivity analysis indicated that Myc potency was highly sensitive to parameters involved in stabilization of Myc and maintenance of the stable form (Table S6). In comparison, other parameters governing the signal transduction in the PI3K pathway had little impact on Myc potency (Table S7). This may partially result from the signaling transduction in the PI3K pathway operating with zero-order ultrasensitivity around the base parameter setting (see Figures S2 and S5 for additional analysis and discussion), which may explain robustness to random perturbation in a signaling cascade [35].

### Effects of Erk and PI3K Signal Patterns on Myc Accumulation

**Figure 2:**
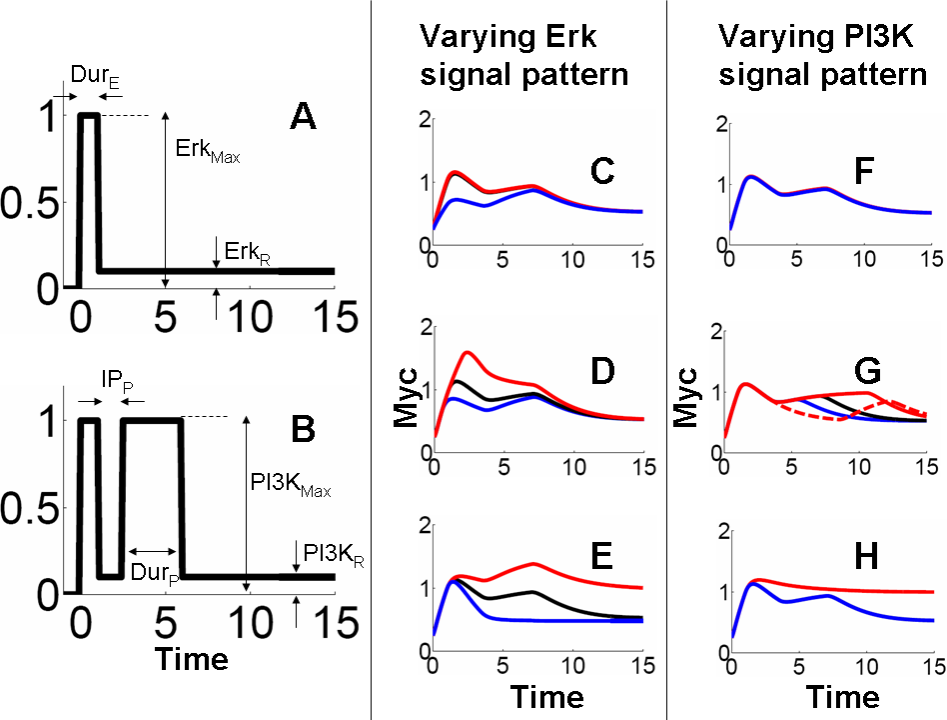
Erk and PI3K signal patterns determine Myc temporal behaviors. For all analyses, black lines represent the base case. (A) The Erk signal was represented with the following parameters: duration (Dur_E_), maximal Erk amplitude (Erk_Max_), and residual Erk level (Erk_R_). (B) The PI3K signal was represented with the following parameters: duration (Dur_P_), maximal PI3K amplitude (PI3K_Max_), residual PI3K level (PI3K_R_), and the time interval between the two peaks of PI3K (IP_P_). The first peak of the PI3K was not considered, since its variations did not have a big impact. (C) Myc accumulation was insensitive to Erk_Max_. Fivefold increase in Erk_Max_ resulted in little change in Myc (red line) in comparison to the base case (black line), whereas fivefold decrease in Erk_Max_ resulted in light reduction in the main peak of Myc (blue line). (D) Doubling (red line) or halving (blue line) Dur_E_ leads to significant change in the initial peak of Myc accumulation. (E) Myc was sensitive to Erk_R_. The base value of Erk_R_ was 10 percent of Erk_Max_ (black line). A small increase in Erk_R_ (20% of Erk_Max_) resulted in excessive Myc accumulation (red line). When Erk was completely removed (Erk_R_ = 0), Myc responded only to the initial, transient Erk pulse and became unresponsive to the PI3K signal (blue line). (F) Myc accumulation was insensitive to PI3K_Max_. Fivefold increase (red line) or decrease (blue line) in PI3K_Max_ resulted in little change in Myc accumulation. (G) The 2^nd^ PI3K peak determined generation and maintenance of Myc hump. Doubling (red line) or halving (blue line) the duration of the second PI3K peak led to approximately twofold change in the Myc hump duration. Increasing IP_P_ from 3 hours to 8 hours delayed the timing of the second rise in Myc accumulation (red dotted line). (H) A slight increase (20% of PI3K_Max_) in PI3K_R_ from the base value (10% of PI3K_Max_) resulted in excessive Myc accumulation (red line). However, complete removal of PI3K_R_ did not change Myc accumulation significantly (blue line overlapping with black line).

Erk and PI3K activation patterns, which determine the temporal dynamics of Myc, may vary significantly under different growth conditions and in different cell lines (Table S1 and S2). Here we investigated how Myc potency responds to varying patterns of Erk and PI3K signals. Whenever possible, model predictions were compared with existing experimental observations. When the latter are unavailable, our model predictions may serve as testable hypothesis for future experiments, which in turn can further constrain our model. As shown in Figure 2, we quantitatively represented input signals of Erk and PI3K with the following parameters: duration (Dur_E_ and Dur_P_), maximal amplitude (Erk_Max_ and PI3K_Max_), and residual level (Erk_R_ and PI3K_R_). For PI3K, we used an additional parameter to describe the time interval between the two peaks (IP_P_).

Our analysis predicted Myc accumulation to be insensitive to further increase in Erk amplitude. A fivefold increase in Erk_Max_ caused little change in Myc accumulation (Figure 2C). A fivefold decrease in Erk_Max_, however, predicted a slight but discernable decrease in Myc accumulation. These results indicated that the base case of Erk was operating at saturation. As a result, this behavior enabled the system to be insensitive to minor changes in Erk amplitude, unless the Erk amplitude became sufficiently small. In comparison, the Myc potency was much more sensitive to the duration of Erk pulse: excessive accumulation of Myc was also observed when the duration of Erk was doubled (red line in Figure 2D). Halving Erk duration resulted in significant reduction in the initial peak of Myc.

Myc potency was sensitive to the residual Erk level (Erk_R_). Without it (Erk_R_ = 0), the total Myc level quickly reduced to a low level following the Erk pulse (blue line in Figure 2E). Conversely, a mere twofold increase in Erk_R_ from the base value ( = 10% of Erk_Max_) led to excessive Myc accumulation (red line in Figure 2E). These results highlighted the importance of Erk_R_ in fine-tuning total Myc accumulation. In particular, Erk_R_ was important for maintaining sufficient Myc level before the arrival of the second PI3K pulse, by providing a moderate rate of Myc stabilization. In a more extreme case where the Erk signal was completely removed, no Myc accumulation was observed (data not shown). These results may provide a mechanistic explanation for differential phenotypic responses to varying residual level of Erk [21]. In PC12 cells proliferation was correlated with low residual level of Erk, while high residual level of Erk was observed for differentiation. Based on our simulations, we suggest that differential regulation of Myc accumulation may be involved in determining these diverging phenotypic behaviors of these cells. This prediction can be tested by further experiments.

Similarly, Myc accumulation was insensitive to the maximum amplitude of PI3K (PI3K_Max_), but much more sensitive to its residual level (PI3K_R_) and temporal features, including duration of the 2^nd^ peak (Dur_P_) and time interval between the two peaks (IP_P_). Five-fold increase or decrease in PI3K_Max_ resulted in little change in Myc accumulation (Figure 2F). However, doubling or halving the duration of the 2^nd^ PI3K peak caused an approximately two fold change in the duration of the Myc hump (Figure 2G). Complete removal of the 2^nd^ PI3K peak eliminated the Myc hump (Figure S3A). This indicates that the 2^nd^ PI3K peak was primarily responsible for generating and maintaining the hump in Myc activation. These results are consistent with recent experimental data: removal of the 2^nd^ PI3K peak by using a PI3K inhibitor [16] or by acid washing [27] drastically reduced total Myc accumulation. Given this role of the 2^nd^ PI3K peak, the time interval between the two peaks of PI3K was critical for determining Myc accumulation pattern (dotted red line in Figure 2G). This is highlighted by a variable time interval across different cell lines or growth conditions. For example, the PI3K inter-peak delay is 3∼4 hours in HepG2 cells [25] but approximately 8 hours in NIH 3T3 cells [16],[25]. Our model was able to account for Myc accumulation pattern in both conditions by varying only the time-interval (either 3 hrs or 8 hours) between the two peaks of PI3K (Figure S3B).

Another sensitive parameter of PI3K was its residual level. A mere two-fold increase in the residual level from the base case (10% of PI3K_Max_), resulted in excessive increase in Myc level (red line in Figure 2H), consistent with an experimental study where exogenous Akt expression induced significantly increased Myc protein levels [36]. Interestingly, however, PI3K_R _below a certain threshold level did not have much impact on Myc accumulation (blue line overlapping with black line in Figure 2H). Such threshold effect is due to the ultrasensitivity in the PI3K signaling cascade (Figures S2 and 5). If the change in the PI3K residual level triggers a digital switching behavior, it can cause a large change in the output (black to red lines in Figure 2H). Any change in the residual level outside the ultrasensitive region will not cause any significant output change.

The results in Figure 2 suggest that Myc accumulation was insensitive to changes in the maximum amplitude of Erk and PI3K signals (Figure 2C and 2F), but much more sensitive to their temporal features such as duration and inter-peak time delay, and their residual values (Figure 2D, 2E, 2G, and 2H). This occurred because the maximum amplitudes of Erk and PI3K pulses were at their saturation level. That is, when the Erk pulse is sufficiently strong, Myc is almost completely converted into Myc^Ser62^; strong PI3K pulses block further phosphorylation of Myc^Ser62^ to Myc^Thr58^. If so, this mechanism will allow cells to resist further changes in Erk and PI3K amplitudes. Such resistance (or insensitivity) to amplitude changes (or fluctuations) of Erk and PI3K may underlie precise control of signal transduction by Myc, given its role as a key regulator of downstream cellular events. That is, dysregulation of Myc activities, which is a signature of various cancers, may have detrimental consequences [37]. To prevent such dysregulation, activation or deactivation cues must be transmitted and integrated precisely to regulate Myc accumulation. We note that this noise-resistance, which we define as insensitivity to the changes in Erk or PI3K level in individual cells, requires the maximum amplitudes of Erk and PI3K to be sufficiently large. If their amplitudes and residual values are set 10 fold lower, the Myc accumulation becomes much more sensitive to perturbations around the new base values.

### The Dual-Kinase Motif as a Generic Signal Integrator

**Figure 3:**
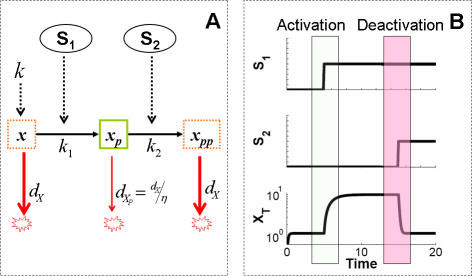
Dual-kinase module as a signal integrator. (A) The dual-kinase mechanism. *S_1_* and *S_2_* determine gain and loss of X stability by sequential phosphorylation, which in turn control the total amount of the target protein (*x_T_* = *x*+*x_P_*+*x_PP_*). *k_1_* and *k_2_* are the rate constants for phosphorylation by S1 and S2, respectively. *d_X_* and 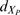 are degradation rate constants of the unstable (*X* or *X_pp_*) and stable (*X_p_*) forms of X. (B) Given sufficiently strong input signals S_1_ and S_2_, the dual kinase mechanism integrates upstream activating signal S_1_ to turn on, and deactivating signal S_2_ to turn off. The time delay between the two signals controls the duration of activation.

The Erk and PI3K pathways that control Myc protein turnover are conserved in yeast [15], and may represent a general post-translational strategy in natural signaling pathways [38]–[44] (Table 1). For instance, β-catenin stability is regulated by casein kinase Iα (CKIα) and Gsk3β [42],[44]. Similarly, an unknown kinase and Gsk3β coordinate to modulate microtubule (MT) stabilizing activity [43]. These examples consist of a dual-kinase motif that integrates two independent input signals (Figure 3A). In this motif, X represents the unphosphorylated effector protein, which is unstable. It can be stabilized by kinase S_1_ through phosphorylation (becoming Xp) and subsequently destabilized by kinase S_2_ through additional phosphorylation (becoming Xpp), as shown in Figure 3B.

**Table 1:** Examples of protein modulation by sequential phosphorylation

	Enzymes	Module function	References
Myc	Erk, PI3K	Protein stabilization	[12]–[14]
Fos	Erk	Protein stabilization	[38]
Jun	JNK, Erk	Protein stabilization	[39]
Β-catenin	CKIα, Gsk3β	Protein stabilization	[42],[44]
LPR6	Gsk3 β, CK1	Axin binding	[40]
CDC25A	B-Cdk1, ATM-Chk2	Stabilization	[41]

The wide presence of this motif suggests its potential advantages for cellular signal processing. To gain insights into this issue, we developed a simplified model to analyze dynamics of the dual-kinase motif (see Model and Methods). In the model, we treated the two inputs of the system (Figure 3A) as independent, decoupled upstream signals, since most of the dual-kinase motifs found in nature often integrate independent upstream signals. Using this model, we aimed to explore what properties of this basic motif may underlie the dynamics observed for Myc regulation, and what advantages these properties may confer in cellular signaling.

**Figure 4:**
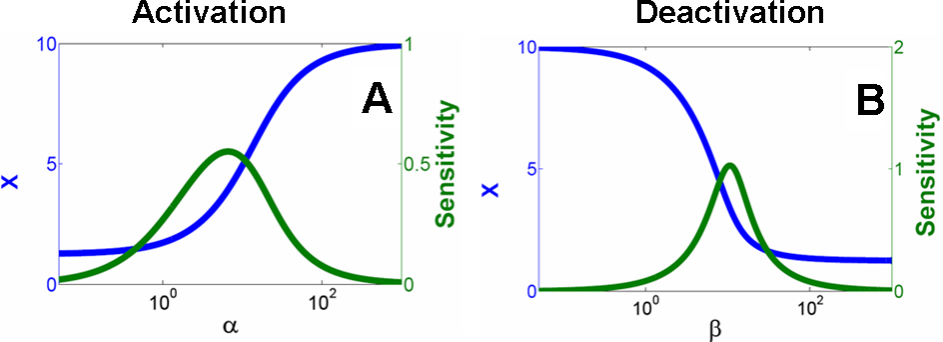
System sensitivity to input signal perturbations. (A) At a given synthesis rate constant (*κ* = 10), the maximal activated level of X at the steady-state (X_ss_) can be modulated by *α*. For small or large *α*, sensitivity (defined as ln *X*/ln *α*) was minimal, while it was the greatest at intermediate *α* values. We assumed 0 for *β* to allow decoupling of activation from deactivation. (B) Deactivation from the high state depended on *β* at a given *κ*. The system was initially driven to its high state by assuming a large *α* (10,000). Sensitivity was minimal for small or large *β*, and was the greatest at intermediate *β* values.

To characterize the dual-kinase motif, we first examined dose response of the system with respect to the two inputs S_1_ and S_2_. Our results indicated that system activation (through phosphorylation by S_1_) was sensitive to input variations at an intermediate *α* value (Figure 4A). In contrast, it was insensitive to input variations at either high or low *α* values (green curve). Similar insensitivity (or noise-resistance) at either strong or weak input signals was observed for system deactivation (Figure 4B) for varying β. These results provide a mechanistic explanation for the insensitivity of Myc potency to input signal strengths (Figure 2C and 2F).

**Figure 5:**
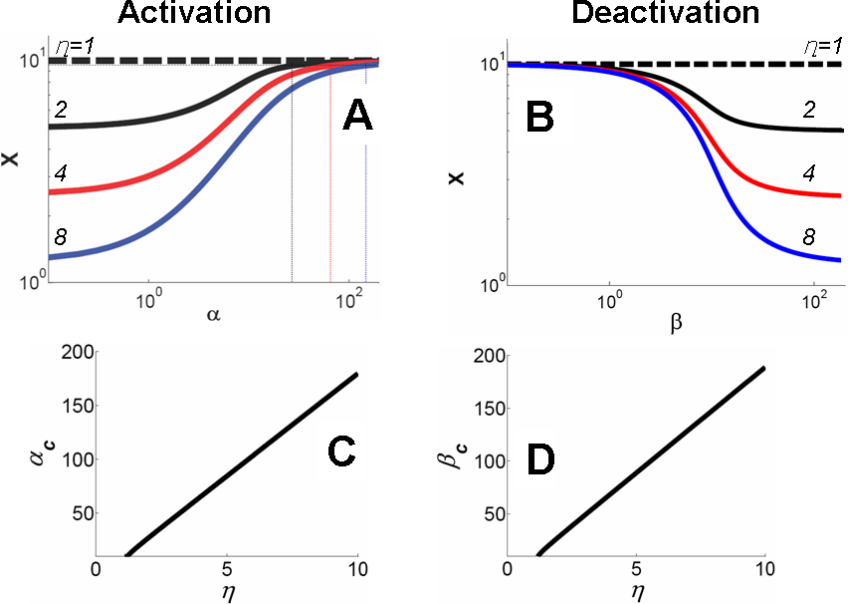
Dynamic range of output activation and deactivation. (A) The dynamic range for activation was *η* because: X_ss_≈*κ/η* as *α*→0; X_ss_≈*κ* as *α*→∞. For a given *η*, we define a critical value *α_C_* that corresponds to an X_SS_ = 95% of the maximal value. If *α*>*α_C_*, fluctuations in X_ss_ due to fluctuations in *α* would be smaller than 5%. Here we consider system activation in this parameter range as effectively noise-resistant. Similar to analyses in Figure 4, we assumed 0 for *β* and a large value (10,000) for *α*, which allowed analyzing dynamic range for activation and deactivation independently. (B) Given a sufficiently large *α*, the dynamic range for system deactivation was also *η* because: X_ss_≈*κ* as *β*→0; X_ss_≈*κ/η* as *β*→∞. For a given *η*, we define a critical value *β_C_* that corresponds to X_ss_ within 5% of its minimal value. Similar to (A), we consider system deactivation to be effectively noise-resistant for *β>β_C_*. (C) *α_C_* increased with *η* almost linearly. (D) *β_C_* increased with *η* almost linearly.

Another salient feature of the dual kinase motif was the stabilization of X, which could be captured by the stabilization efficiency (*η*), or the ratio between the degradation rate constant of the unstable form and that of the stable form. Our analysis indicated that the stabilization efficiency determines the dynamic range of the output X. In response to S_1_, the upper bound of output was set by the synthesis rate of X (*κ*) and was asymptotically approached as the signal strength (α) increasedh (Figure 5A). The lower bound of the output, however, was set by *κ/η*, which corresponded to the basal level of X in the absence of S_1_. Thus, *η* directly set the dynamic range of the output (*κ/η*∼*κ*). Similar dependence was also applied to deactivation of X by S_2_ (Figure 5B). Given saturating activation by S_1_, the dynamic range for deactivation by S_2_ increased with increasing *η,* allowing the dynamic range to be flexible. The system approached the basal output level (*κ/η*) with an increasing strength of S_2_ (β→∞).

These results highlight two appealing features of the dual kinase motif. First, differential stability control on effector protein isoforms enables flexible modulation of the output dynamic range. This dynamic range can be fully exploited if the signal strengths are sufficiently large. Second, sufficiently strong signals will also result in desensitization of the system output to minor fluctuations in the levels of these signals.

While advantageous, however, increase in noise-resistance and dynamic range comes with increasing metabolic cost. On one hand, increasing destabilization of X or Xpp is associated with increasing metabolic cost. On the other, this will also require stronger input signals to fully exploit the increased dynamic range and to achieve noise-resistance, creating another metabolic burden as characterized by *α* and *β*. To quantify this effect, we define a critical *α* value (*α_C_*), which corresponds to a steady-state X (Xss) value at 95% of the maximum X (for *α*→*∞)*. If the input signal would fluctuate in the range of *α*>*α_C_*, the resulting output fluctuation would never exceed 5% (regardless of the magnitude of input signal fluctuation). Here we can consider system activation as noise-resistant in this parameter range. With similar reasoning, we define a critical *β_C_*, which corresponds to an Xss value within 5% of the minimum X (for *β*→*∞*). *α_C_* and *β_C_* thus determine the minimal signal strengths required to achieve noise-resistance in system activation or deactivation. As shown Figure 5C and 5D, the greater the stabilization efficiency was (larger *η*), the heavier would be the corresponding metabolic burden (larger *α_C_* or *β_C_*) required to achieve noise-resistance. Insufficient input signal strength would either fail to generate response or fall into the sensitive range of the dose response curve (Figure 4).

Here we demonstrate that modulation of Myc stability by sequential phosphorylation enables Myc to precisely sense and integrate upstream Erk and PI3K signals. Such regulation is likely critical to cell fate decisions. Our analysis indicates that, when operating with appropriate parameters, this mechanism enables the temporal features, instead of maximum amplitudes, of the upstream signals to precisely modulate Myc accumulation. Supporting this notion, dynamics of a minimal dual-kinase motif provide direct, intuitive explanation for the key sensitivity properties of Myc output in the full model. In this work, we have limited our study to the well-defined post-translational control of Myc. It is possible that robust control of Myc accumulation is facilitated by additional mechanisms, including Myc stabilization by a signal in the carboxy-terminus of Myc [45] and Myc sequestration for degradation [46],[47]. Myc modulation is also tuned by regulations at other levels including post-transcription [10] and translation [48], along with feedback control [9]. Furthermore, the activities of Pin1 and PP2A, which we assumed to be abundant and not rate-limiting, may further contribute to more complex Myc dynamics, as seen in various cancers [49]–[52].

As Myc is often deregulated in cancers, quantitative understanding of the mechanisms for Myc regulation may be helpful for developing novel strategies for cancer treatment. Myc stabilization processes consist of two temporally coordinated events: Myc stabilization by Erk and prevention of Myc degradation by PI3K. While the significance of Myc degradation by the second PI3K activity has been suggested in cell proliferation [16], the extent to which the initial Myc stabilization by Erk contributes to cell proliferation remains unknown. Our model predicts that, for the second round of Myc accumulation, Myc needs to be sufficiently accumulated by Erk_R_ prior to the second PI3K activity (black line in Figure S4). With the PI3K signal fixed, a small increase in Erk_R_ is predicted to result in a significant increase in Myc accumulation pattern (red line in Figure S4). In contrast, removal of Erk_R_ renders Myc unresponsive to the PI3K signal (blue line in Figure S4). This is due to the sequential nature of the Myc stabilization processes, where Erk activity must precede PI3K activity. In other words, while Erk ‘primes’ Myc activity, PI3K ‘fine-tunes’ Myc accumulation.

The priming ability of Erk for Myc modulation may play a critical role in distinct responses to different stimulations. Studies have shown that PC12 cells can be induced to undergo differentiation or proliferation in response to NGF or EGF [49],[50], and the residual Erk level may be responsible for these differential cellular responses [21]. Our analysis suggests that the ability of Erk to modulate these cellular responses is through modulation of Myc accumulation. For EGF stimulation, the low residual Erk level may induce proliferation by weakly priming Myc (black line in Figure S4). In contrast, the high residual Erk level upon NGF stimulation may lead to a significant increase in Myc, inducing differentiation (red line in Figure S4). This notion can be experimentally tested by simultaneous time-course measurements of the input signals Erk and PI3K, and the output Myc protein. Also, the input signals can be independently controlled by inhibitor drugs [51], inducible systems, or siRNA molecules targeting the MAPK or PI3K pathways [52].

The assumed saturation of the input signals in the base model can also be experimentally tested. Our simulations indicate that the assumed saturation is a necessary condition for the overall robustness of Myc to parameters. This serves as an interesting question to explore experimentally. Also, as detailed in Figure S5, some constituent reactions in the PI3K pathway (e.g., the Ph-dePh cycles) have not been well-characterized at the quantitative level. Our additional model predictions on how the overall response of Gsk3β to PI3K depends on sensitivity characteristics of individual stages can serve as further targets for experimental tests.

The analysis of the Myc stabilization mechanism reveals a regulatory network motif that may be ubiquitously used in nature. Network motifs are small, recurring cellular regulatory networks, identified and characterized by their shared architectures and functions among diverse organisms. Well-known examples include feedback regulations, feed-forward loops, and their derivatives (see [53]–[56] for review). Here we suggest that the dual-kinase motif represents another example with distinctive features.

The dual-kinase motif is similar to a well-studied phosphorylation-dephosphorylation enzymatic motif of protein modification. In both motifs, protein modification events occur sequentially, and the current state of the protein hinges upon its previous state. Given appropriate input signals and parameters, the sensitivity and amplitude of the output response can be precisely controlled [57]. The dual-kinase motif differs from the phosphorylation-dephosphorylation one, however, in that protein modification process is irreversible. Once phosphorylated, the stabilized protein cannot return to its initial state, but is targeted for degradation upon further modification. This distinctive characteristic contributes to additional features of the dual-kinase motif: sequential signal integration of multiple inputs and, correspondingly, flexible dynamic range for the output governed by protein stability modulation.

## Model and Methods

### Myc Regulation by Erk and PI3K

Based on the reaction network outlined in Figure 1A, we developed a kinetic mathematical model in *Dynetica*, a graphics-based, integrated simulation platform [58]. We further simplified the model by lumping the sequential destabilization processes together into the phosphorylation rate constant for Myc^Thr58^ (Figure S1). This simplification was based on the observation that Pin1 and PP2A activation is not rate-limiting during cell cycle entry [59],[60]. Explicitly accounting for the sequential events by Pin1 and PP2A did not have significant impact on Myc accumulation. In addition, we assumed that the change in transactivation capacity due to stability control [14] is not significant, since Myc^Ser62^ is much more predominant than Myc^Thr58^. Based on experimental data, we assumed phosphorylation of Myc at Ser62 or Thr58 to be much more significant than dephosphorylation. This results in sequential, irreversible Myc stabilization. However, we accounted specifically for the differential degradation dynamics of protein isoforms in this model.

To establish a framework that facilitates investigation of Myc modulation by its upstream signals, Erk and PI3K, we built the model with Erk and PI3K as the inputs and Myc as the output. Despite the extensive interactions between the MAPK and PI3K pathways, we decoupled Erk and PI3K signals and simplified them as a single or double rectangular pulses, respectively (Figure 1B). Such decoupling of these signals was driven by the objective of our study: to characterize Myc's response to various input signal patterns. Since activation of Erk and PI3K is specific to cell lines and stimulants (as shown in Table S1 and S2), and is mediated by multiple signaling pathways including Ras, Rac, or Rap [61]–[64], it is not clear to what extent these signaling pathways contribute to Erk and PI3K activation patterns. Furthermore, the Erk and PI3K pathways that control Myc protein turnover are a conserved motif found in both mammalian and yeast systems and such control motif has been speculated in many other protein stabilization processes [15]. In many of these processes, input signals are not triggered by a single protein. To set up a framework for a more general Myc stabilization process, we decoupled the two signals from each other and from their upstream network regulation and assumed their effects on Erk and PI3K in the decoupled inputs. This allowed analyzing Myc's response to variations in Erk and PI3K independently.

Based on experimental observations, we approximated input signals as rectangular pulses with three parameters: duration, the maximum level, and the residual level. To describe two-peak PI3K activation, we introduced another parameter, inter-peak delay. More sophisticated representations (for example, sinusoidal pulses) give similar results (data not shown). Although we focused on the two-peak activation of PI3K in the base model, the modeling framework can be extended to study other patterns of PI3K signals (such as a single peak pattern) by varying duration, steady-state values, or inter-peak delay (for example, see Figure S3).

As detailed in Figure S1 and Tables S4, most reaction mechanisms and base model parameters were derived from experimental results in mouse or human cells. Others were carefully obtained or estimated from previous theoretical studies [65]–[67]. To test the effect of uncertainty in these parameter values, we carried out parametric sensitivity analysis by increasing or decreasing each parameter by 10 fold of its base value while keeping the others constant (Figure S6). To quantify Myc's response to these changes, we used ‘potency’ or the shaded area under the Myc temporal profile curve (Figure 1B). This quantification method was previously used to characterize potency of transient activation of signaling protein [68]. The parametric sensitivity analysis was performed in *Matlab*.

### Modeling a Generic Dual Kinase Motif

Based on the connectivity in Figure 3A, we modeled the dual kinase motif using three highly simplified ordinary differential equations (ODEs), as presented in a dimensionless form:
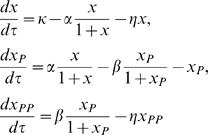
where *x*, *x*_P_, and *x_PP_* are the concentrations of the three forms of a molecule X; *τ* is an independent variable, time; *κ* describes the synthesis of X (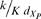); *α* is the activation efficiency by S_1_ (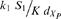); β is the deactivation efficiency by S_2 _(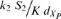); and *η* is the ratio of unstable protein to stable protein (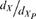), or *stabilization efficiency*. Without loss of generality and for simplification, we assumed that *x* and *x_pp_* had the same stability.

Similar to Myc regulation, we used the total effector concentration X ( = *x*+*x_P_*+*x_PP_*) to represent the system output. As shown by a typical simulation (Figure 2B), this module enables integration of two signals by the effector module. Drawing analogy to electric signal processing, the output can be considered a combination of ‘NOT’ and ‘AND’ operators, which defines a pulse of output.

## Supporting Information

Figure S1 Detailed reaction diagram for Myc protein stabilization.
(0.22 MB TIF)

Figure S2 Modeling a phosphorylation-dephosphorylation cycle. An enzymatic modification cycle of Gsk3β between phosphorylated and dephosphorylated states (A) is mathematically modeled (B). *k* and *k_GP_* are rate constants for phosphorylation and dephosphorylation, and *K* is the Michaelis-Menten constant. Protein conversion is ultrasensitive near γ = 1, for a sufficiently small Michaelis-Menten constant. The sensitivity becomes weaker as *K* is increased. Time-course simulation results at varying values show the dependence of conversion on the rates of phosphorylation and dephosphorylation (C). Protein conversion becomes ultrasensitive near α = 1 for a sufficiently small Michaelis-Menten constant, while the sensitivity becomes weaker as *K* is increased.
(0.12 MB TIF)

Figure S3 Impact of varying PI3K inputs on Myc accumulation. (A) A single peak of Myc is predicted if the second round of PI3K activity is removed. This results in reduced Myc accumulation compared to the wild-type. (B) Increased inter-peak time delay of PI3K (from 3 to 8 hours) results in wider separation between the two peaks of Myc, and the resulting Myc accumulation is less than the wild-type.
(0.11 MB TIF)

Figure S4 Erk ‘primes’ Myc activity, and PI3K ‘fine-tunes’ Myc accumulation level. With the PI3K signal fixed, different residual Erk level leads to differential Myc accumulation by the second PI3K activity. The base value of the residual Erk level (ErkR) was 10 percent of maximal Erk level (black line). For increased level of ErkR (20%), the second PI3K activity increased Myc accumulation level significantly (red line). When ErkR was completely removed, Myc became unresponsive to the PI3K signal (blue line).
(0.09 MB TIF)

Figure S5 The overall ultrasensitivity arises from the input/output response in each level and across different levels down the cascade. (A) The Akt Ph-dePh cycle (in response to PI3K) can be either graded (red line) or ultrasensitive (blue line) depending on the Michaelis-Menten constants. (B) Both types of PI3K-Akt responses can lead to ultrasensitive PI3K-Gsk3β responses (both red and blue), if the Akt-Gsk3β response remains ultrasensitive. (C) If Akt-Gsk3β response is not ultrasensitive, the overall PI3K-Gsk3β remains ultrasensitive if PI3K-Akt response is ultrasensitive, but may lose ultrasensitivity if PI3K-Akt response is not ultrasensitive. Note that here we have assumed that the output from the first step (Akt_P_) has an appropriate dynamic range that “matches” the input of the second step. The dependence of the overall sensitivity of the PI3K-Gsk3β response will likely be much more complex if this matching condition is not satisfied.
(0.22 MB TIF)

Table S1 Erk signal pattern
(0.05 MB DOC)

Table S2 PI3K signal pattern
(0.05 MB DOC)

Table S3 Myc signal pattern
(0.03 MB DOC)

Table S4 Reaction kinetics
(0.09 MB DOC)

Table S5 Base model parameters and notes
(0.08 MB DOC)

Table S6 Parametric Sensitivity
(0.03 MB DOC)

Table S7 Parametric Sensitivity without ultrasensitivity
(0.03 MB DOC)

Text S1 (0.05 MB DOC)
