# Supplementary figures and images for: Sensing and Integration of Erk and PI3K Signals by Myc
Source: PLoS Comput Biol. 2008 Feb 29;4(2):e1000013. doi: 10.1371/journal.pcbi.1000013 (PMC2265471; doi:10.1371/journal.pcbi.1000013)

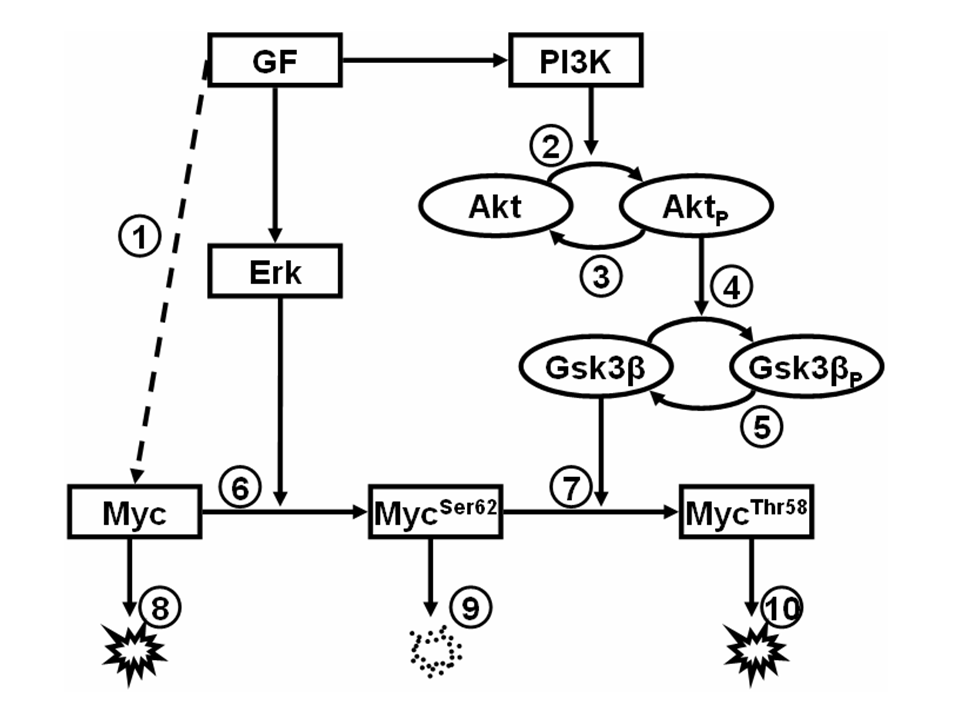

Supplement: Figure S1 — Detailed reaction diagram for Myc protein stabilization. (0.22 MB TIF) [file pcbi.1000013.s001.tif]

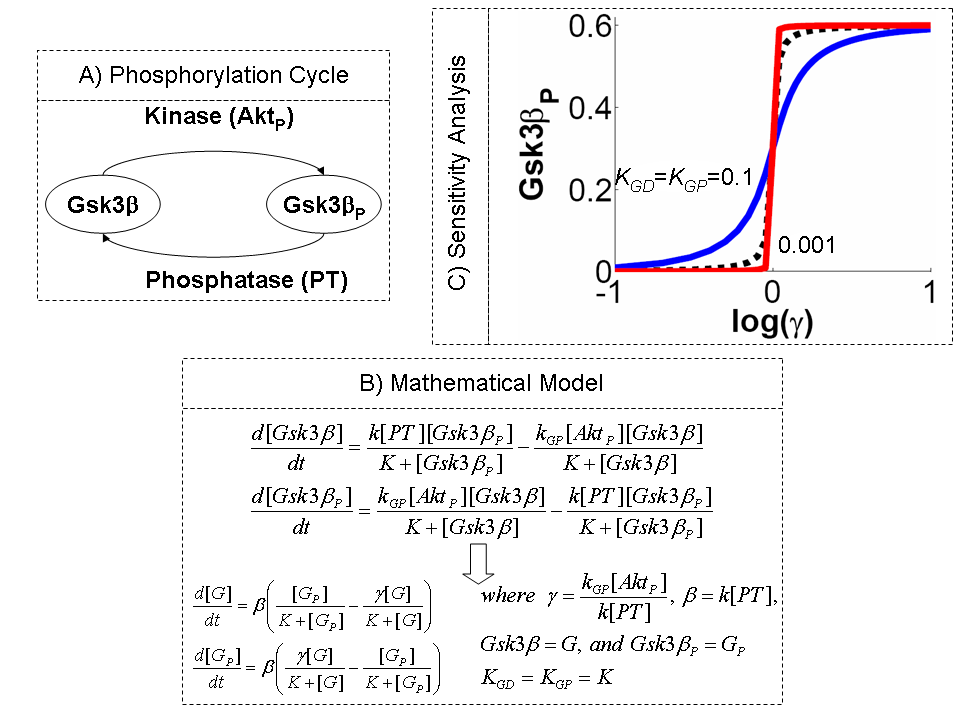

Supplement: Figure S2 — Modeling a phosphorylation-dephosphorylation cycle. An enzymatic modification cycle of Gsk3β between phosphorylated and dephosphorylated states (A) is mathematically modeled (B). k and kGP are rate constants for phosphorylation and dephosphorylation, and K is the Michaelis-Menten constant. Protein conversion is ultrasensitive near γ = 1, for a sufficiently small Michaelis-Menten constant. The sensitivity becomes weaker as K is increased. Time-course simulation results at varying values show the dependence of conversion on the rates of phosphorylation and dephosphorylation (C). Protein conversion becomes ultrasensitive near α = 1 for a sufficiently small Michaelis-Menten constant, while the sensitivity becomes weaker as K is increased. (0.12 MB TIF) [file pcbi.1000013.s002.tif]

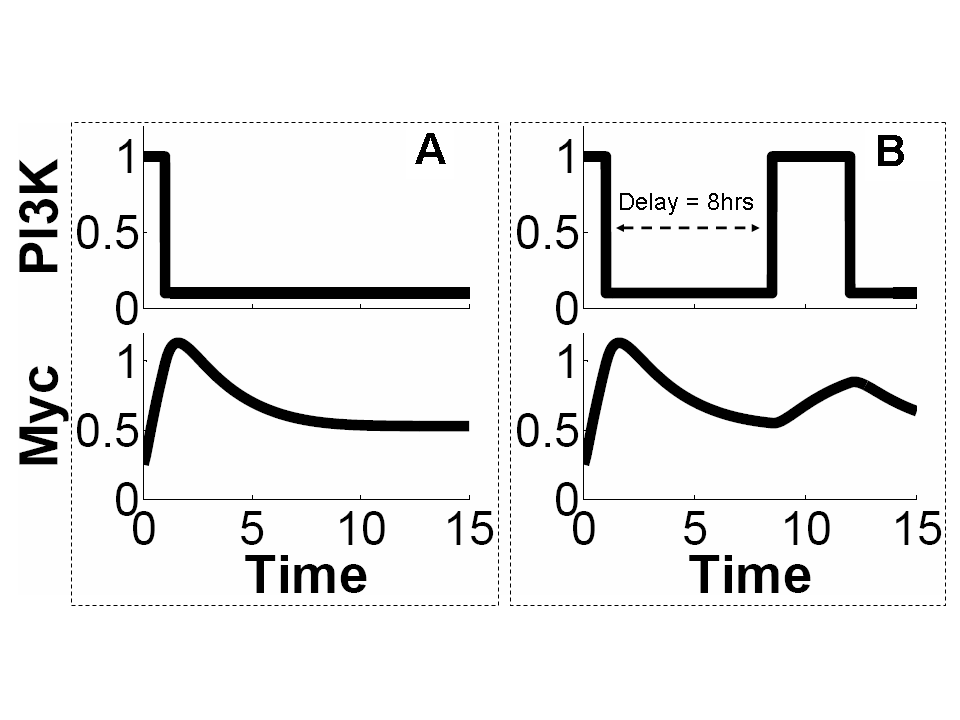

Supplement: Figure S3 — Impact of varying PI3K inputs on Myc accumulation. (A) A single peak of Myc is predicted if the second round of PI3K activity is removed. This results in reduced Myc accumulation compared to the wild-type. (B) Increased inter-peak time delay of PI3K (from 3 to 8 hours) results in wider separation between the two peaks of Myc, and the resulting Myc accumulation is less than the wild-type. (0.11 MB TIF) [file pcbi.1000013.s003.tif]

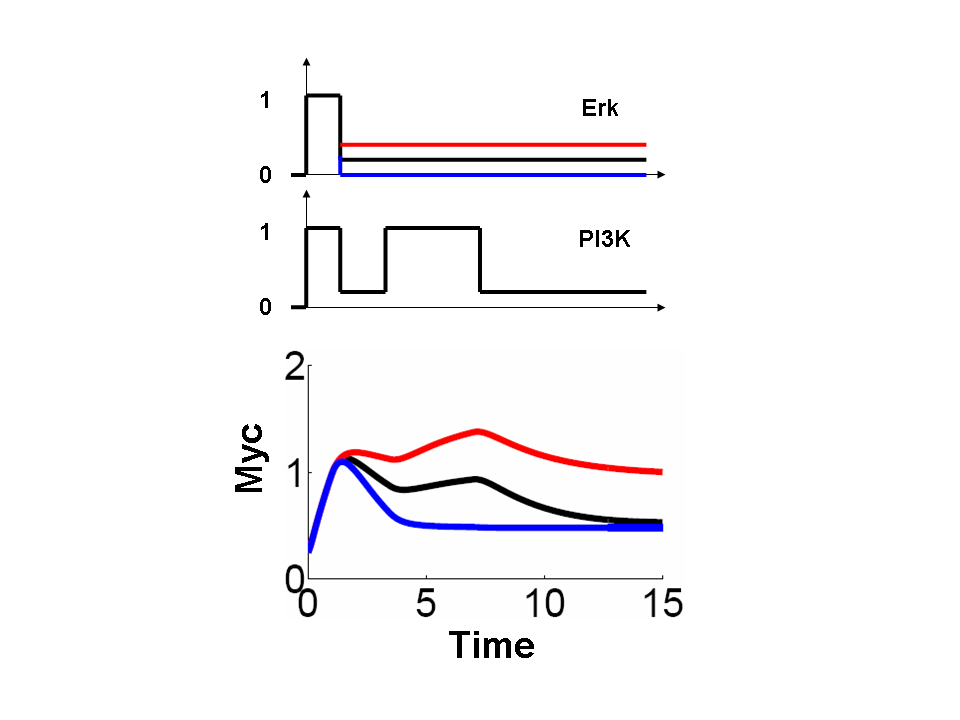

Supplement: Figure S4 — Erk ‘primes’ Myc activity, and PI3K ‘fine-tunes’ Myc accumulation level. With the PI3K signal fixed, different residual Erk level leads to differential Myc accumulation by the second PI3K activity. The base value of the residual Erk level (ErkR) was 10 percent of maximal Erk level (black line). For increased level of ErkR (20%), the second PI3K activity increased Myc accumulation level significantly (red line). When ErkR was completely removed, Myc became unresponsive to the PI3K signal (blue line). (0.09 MB TIF) [file pcbi.1000013.s004.tif]

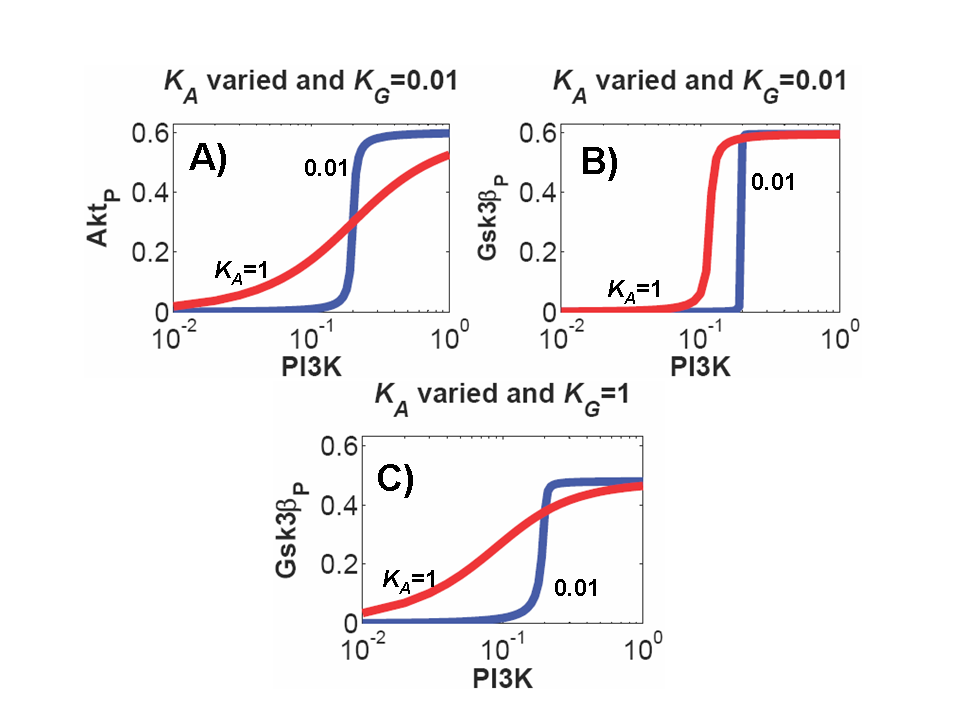

Supplement: Figure S5 — The overall ultrasensitivity arises from the input/output response in each level and across different levels down the cascade. (A) The Akt Ph-dePh cycle (in response to PI3K) can be either graded (red line) or ultrasensitive (blue line) depending on the Michaelis-Menten constants. (B) Both types of PI3K-Akt responses can lead to ultrasensitive PI3K-Gsk3β responses (both red and blue), if the Akt-Gsk3β response remains ultrasensitive. (C) If Akt-Gsk3β response is not ultrasensitive, the overall PI3K-Gsk3β remains ultrasensitive if PI3K-Akt response is ultrasensitive, but may lose ultrasensitivity if PI3K-Akt response is not ultrasensitive. Note that here we have assumed that the output from the first step (AktP) has an appropriate dynamic range that “matches” the input of the second step. The dependence of the overall sensitivity of the PI3K-Gsk3β response will likely be much more complex if this matching condition is not satisfied. (0.22 MB TIF) [file pcbi.1000013.s005.tif]
