# Supplementary material for: Sensing and Integration of Erk and PI3K Signals by Myc
Source: PLoS Comput Biol. 2008 Feb 29;4(2):e1000013. doi: 10.1371/journal.pcbi.1000013 (PMC2265471; doi:10.1371/journal.pcbi.1000013)
Supplement: Table S1 — Erk signal pattern (0.05 MB DOC) [file pcbi.1000013.s006.doc]

Table S1: Erk signal pattern (Transient strong activation followed by varying residual

levels)

| **Cell Line** | **Growth Factor** | **Cell outcome** | **References** |
| --- | --- | --- | --- |
| PC12 | EGF | Proliferation | [1,2] |
| PC12 | NGF | Differentiation | [1-3] |
| CCL39 | Thrombin | Proliferation | [4] |
| Swiss 3T3 | PDGF | Proliferation | [5] |
| Swiss 3T3 | EGF | No proliferation | [5] |

**References:**

1. Sasagawa S, Ozaki Y, Fujita K, Kuroda S (2005) Prediction and validation of the distinct dynamics of transient and sustained ERK activation. Nature Cell Biology 7: 365-U331.

2. Traverse S, Seedorf K, Paterson H, Marshall CJ, Cohen P, et al. (1994) EGF triggers neuronal differentiation of PC12 cells that overexpress the EGF receptor. Current Biology 4: 694-701.

3. Yaka R, Gamliel A, Gurwitz D, Stein R (1998) NGF induces transient but not sustained activation of ERK in PC12 mutant cells incapable of differentiating. Journal of Cellular Biochemistry 70: 425-432.

4. Balmanno K, Cook SJ (1999) Sustained MAP kinase activation is required for the expression of cyclin D1, p21Cip1 and a subset of AP-1 proteins in CCL39 cells. Oncogene 18: 3085-3097.

5. Murphy LO, Smith S, Chen RH, Fingar DC, Blenis J (2002) Molecular interpretation of ERK signal duration by immediate early gene products. Nature Cell Biology 4: 556-564.
