# Supplementary material for: Sensing and Integration of Erk and PI3K Signals by Myc
Source: PLoS Comput Biol. 2008 Feb 29;4(2):e1000013. doi: 10.1371/journal.pcbi.1000013 (PMC2265471; doi:10.1371/journal.pcbi.1000013)
Supplement: Table S2 — PI3K signal pattern (0.05 MB DOC) [file pcbi.1000013.s007.doc]

Table S2: PI3K signal pattern

|  | **Cell line** | **Growth Factor** | **Cell outcome** | **Inter-peak delay (hrs)** | **References** |
| --- | --- | --- | --- | --- | --- |
| Single peak | PVSM | PDGF | Proliferation or  migration | N/A | [1]* |
|  | PVSM | TGFα | Proliferation | N/A | [1]* |
|  | PVSM | Thrombin | Proliferation | N/A | [1]* |
|  | U-2OS | FCS | Proliferation | N/A | [2] |
| Double peaks | NIH 3T3 | FBS | Proliferation | 8 | [3] |
|  | HepG2 | PDGF | Proliferation | 5 | [4] |
|  | WI38 | PDGF | Proliferation | 3 | [2] |

* Experimental observations were made only for the first three hours after stimulation. It is possible that cells exhibit a second round of PI3K activity when measured for a longer time under these conditions.

**References:**

1. Goncharova EA, Ammit AJ, Irani C, Carroll RG, Eszterhas AJ, et al. (2002) PI3K is required for proliferation and migration of human pulmonary vascular smooth muscle cells. American Journal of Physiology - Lung Cellular & Molecular Physiology 283: L354-363.

2. Chaussepied M, Ginsberg D (2004) Transcriptional regulation of AKT activation by E2F. Molecular Cell 16: 831-837.

3. Kumar A, Marques M, Carrera AC (2006) Phosphoinositide 3-Kinase Activation in Late G1 Is Required for c-Myc Stabilization and S Phase Entry. Mol Cell Biol 26: 9116-9125.

4. Jones SM, Klinghoffer R, Prestwich GD, Toker A, Kazlauskas A (1999) PDGF induces an early and a late wave of PI 3-kinase activity, and only the late wave is required for progression through G1. Current Biology 9: 512-521.
