# Supplementary material for: Sensing and Integration of Erk and PI3K Signals by Myc
Source: PLoS Comput Biol. 2008 Feb 29;4(2):e1000013. doi: 10.1371/journal.pcbi.1000013 (PMC2265471; doi:10.1371/journal.pcbi.1000013)
Supplement: Table S3 — Myc signal pattern (0.03 MB DOC) [file pcbi.1000013.s008.doc]

Table S3: Myc signal pattern

|  | **Cell Line** | **Growth factor** | **Cell outcome** | **Inter-peak delay (hrs)** | **References** |
| --- | --- | --- | --- | --- | --- |
| Two peaks | NIH 3T3 | FBS | Proliferation | 8 | [1] |
| Overlapping peaks | Primary Human Fibroblast | FBS | Proliferation | N/A | [2] |

**References:**

1. Kumar A, Marques M, Carrera AC (2006) Phosphoinositide 3-Kinase Activation in Late G1 Is Required for c-Myc Stabilization and S Phase Entry. Mol Cell Biol 26: 9116-9125.

2. O'Donnell KA, Wentzel EA, Zeller KI, Dang CV, Mendell JT (2005) c-Myc-regulated microRNAs modulate E2F1 expression. Nature 435: 839-843.
