# Supplementary material for: Sensing and Integration of Erk and PI3K Signals by Myc
Source: PLoS Comput Biol. 2008 Feb 29;4(2):e1000013. doi: 10.1371/journal.pcbi.1000013 (PMC2265471; doi:10.1371/journal.pcbi.1000013)
Supplement: Table S4 — Reaction kinetics (0.09 MB DOC) [file pcbi.1000013.s009.doc]

Table S4. Reaction kinetics

| **Index**  **Kinetics** | **Reaction** | **Kinetics** | **Description** |
| --- | --- | --- | --- |
| 1 |  | $k_{M}[GF]$ | Synthesis of Myc |
| 2 |  | $\frac{k_{AP}[PI3K] [Akt]}{K_{AP} + [Akt]}$ | Akt phosphorylation |
| 3 | $Akt_{p}\xrightarrow{} Akt$ | $\frac{k_{AD} [Akt_{p}]}{K_{AD}+[Akt_{p}]}$ | Akt dephosphorylation |
| 4 |  | $\frac{k_{GP}[Akt_{p}][Gsk3\beta]}{K_{GP}+[Gsk3\beta]}$ | Gsk3β phosphorylation |
| 5 | $Gsk3\beta_{p}\xrightarrow{} Gsk3\beta$ | $\frac{k_{GD}[Gsk3\beta_{p}]}{K_{GD}+[Gsk3\beta_{p}]}$ | Gsk3β dephosphorylation |
| 6 |  | $\frac{k_{MS}[Erk][Myc]}{K_{MS}+[Myc]}$ | Myc phosphorylation at Ser62 |
| 7 |  | $\frac{k_{MT}[Gsk3\beta][Myc^{Ser62}]}{K_{MT}+[Myc^{Ser62}]}$ | Myc phosphorylation at Thr58 |
| 8 | $Myc\xrightarrow{} *$ | $d_{M}[Myc]$ | Unstable Myc Degradation |
| 9 | $Myc^{Ser62}\xrightarrow{}*$ | $d_{MS}[Myc^{Ser62}]$ | MycSer62 degradation |
| 10 | $Myc^{Thr58}\xrightarrow{} *$ | $d_{MT}[Myc^{Thr58}]$ | MycThr58 degradation |

Reaction 1: Synthesis of Myc in response to growth factor stimulation.

The processes of transcription, post-transcription, and translation are lumped together into one reaction with the synthesis rate constant of *k_M_*

Reaction 2 and 3: Akt phosphorylation and dephosphorylation. Akt activation depends on lipid products of PI3K such as PtdIns-3,4,5-P3. Binding of the Pleckstrin homology (PH) domain of Akt with the PI3K lipid products recruits Akt to the plasma membrane, where Akt becomes phosphorylated at two sites. Phosphorylation at serine473 (Akt^Ser473^) is mediated by PI3K-dependent kinase 2 (PDK2), and this process facilitates the second phosphorylation of Akt at theorine308 (Akt^Ser473-Thr308^). Active Akt translocates to either nucleus or cytosol to regulate a number of downstream proteins [1]. Active Akt in cytosol or nucleus is inactivated by protein phosphatases 2A (PP2A) [2] . The phosphorylation processes are simplified as a single step with the reaction rate constant of *k_AP_*. Phosphorylation is mediated by active PI3K, but we assume a constant level of PP2A for dephosphorylation. Therefore, the dependence of dephosphorylation on PP2A is included in the dephosphorylation rate constant *k_AD_.*

Reaction 4 and 5: Gsk3β phosphorylation and dephosphorylation. Glycogen synthase kinase-3 (Gsk3β), the first identified physiological substrate of Akt, is an enzyme that regulates glycogen synthesis. In resting cells Gsk3β is ubiquitously expressed in its active form, and its activity regulates a number of downstream cellular events, including phosphorylation of Myc at theorine58. Active Akt phosphorylates Gsk3β at Ser9 to inhibit its kinase activity. Phosphorylated Gsk3β gets dephosphorylated by PP2A [3]. Phosphorylation process is mediated by active Akt with the reaction rate constant of *k_GP_* while dephosphorylation is PP2A-dependent with the rate constant of *k_GD_*.

Reaction 6: Myc phosphorylation at serine 62. Active Erk mediates phosphorylation of Myc at Ser62, leading to Myc^Ser62^ which is stable and protected from degradation [4]. The amount of Myc and Erk determines how much of Myc becomes phosphorylated with the rate constant of *k_MS_.*

Reaction 7: Myc phosphorylation at threorine58. Phosphorylation at Thr58 mediated by active Gsk3β requires prior phosphorylation at Ser62. Phosphorylation at Thr58 triggers dephosphorylation at Ser62, resulting in degradable Myc^Thr58^ [5]. We assume that phosphorylation at Thr58 and dephosphorylation at Ser62 are instantaneous. Thus, we treat the two events as a single step mediated by active Gsk3β with the rate constant of *k_MT_*.

Reaction 8: Unstable Myc degradation. Before phosphorylation, Myc is a highly unstable protein, with a typical half-life of ~ 20 min [6]. Myc is destroyed by ubiquitin-mediated proteolysis [7]. Further studies have suggested that the F box protein and ubiquitin ligase component Skp2 is the regulator that signals Myc ubiquitylation and turnover [8]. The half-life of 20 min was assumed for unphosphorylated Myc degradation, represented in *d_M_*

Reaction 9: Myc­­^Ser62^ degradation. Once Myc is phosphorylated at Ser62 by Erk, its stability and half-life increases drastically, up to 2 hours. This is reflected in *d_MS_*

Reaction 10: Myc^Thr58^ degradation. When Ser62 alone is phosphorylated, Myc is not ubiquitinated and remains stable. However, once Myc is phosphorylated at Thr58, proteasome-dependent degradation of Myc is mediated by a F-box protein Fbw7, a component of an SCF-class ubiquitin ligase (E3) complex [9,10]. The half-life of phosphorylated Myc at Thr58 (*d_MT_*) is assumed to be the same as *d_M_*.

**References:**

1. Du KY, Tsichlis PN (2005) Regulation of the Akt kinase by interacting proteins. Oncogene 24: 7401-7409.

2. Hatakeyama M, Kimura S, Naka T, Kawasaki T, Yumoto N, et al. (2003) A computational model on the modulation of mitogen-activated protein kinase (MAPK) and Akt pathways in heregulin-induced ErbB signalling. Biochem J 373: 451-463.

3. Pap M, Cooper GM (1998) Role of glycogen synthase kinase-3 in the phosphatidylinositol 3-kinase/Akt cell survival pathway. Journal of Biological Chemistry 273: 19929-19932.

4. Sears R, Nuckolls F, Haura E, Taya Y, Tamai K, et al. (2000) Multiple Ras-dependent phosphorylation pathways regulate Myc protein stability. Genes & Development 14: 2501-2514.

5. Yeh E, Cunningham M, Arnold H, Chasse D, Monteith T, et al. (2004) A signalling pathway controlling c-Myc degradation that impacts oncogenic transformation of human cells. Nature Cell Biology 6: 308-318.

6. Lepique AP, Moraes MS, Rocha KM, Eichler CB, Hajj GNM, et al. (2004) c-Myc protein is stabilized by fibroblast growth factor 2 and destabilized by ACTH to control cell cycle in mouse Y1 adrenocortical cells. J Mol Endocrinol 33: 623-638.

7. Salghetti SE, Kim SY, Tansey WP (1999) Destruction of Myc by ubiquitin-mediated proteolysis: cancer-associated and transforming mutations stabilize Myc. Embo Journal 18: 717-726.

8. Kim SY, Herbst A, Tworkowski KA, Salghetti SE, Tansey WP (2003) Skp2 regulates Myc protein stability and activity. Molecular Cell 11: 1177-1188.

9. Welcker M, Orian A, Jin JP, Grim JA, Harper JW, et al. (2004) The Fbw7 tumor suppressor regulates glycogen synthase kinase 3 phosphorylation-dependent c-Myc protein degradation. Proceedings of the National Academy of Sciences of the United States of America 101: 9085-9090.

10. Yada M, Hatakeyama S, Kamura T, Nishiyama M, Tsunematsu R, et al. (2004) Phosphorylation-dependent degradation of c-Myc is mediated by the F-box protein Fbw7. Embo Journal 23: 2116-2125.
