# Supplementary material for: Sensing and Integration of Erk and PI3K Signals by Myc
Source: PLoS Comput Biol. 2008 Feb 29;4(2):e1000013. doi: 10.1371/journal.pcbi.1000013 (PMC2265471; doi:10.1371/journal.pcbi.1000013)
Supplement: Table S5 — Base model parameters and notes (0.08 MB DOC) [file pcbi.1000013.s010.doc]

Table S5. Base model parameters and notes

| **Rate Constants** | **Values** | **Source** |
| --- | --- | --- |
| *k_M_* | 1 /hr | Constrained by [1] |
| *Erk_Max_^*^* | 1 /hr | Constrained by [2] |
| *PI3K_Max_^**^* | 1 /hr | Constrained by [3] |
| *k_AP_* | 360 / hr | Typical value phosphorylation rate constant is 3600/hr [4] |
| *K_AP_* | 0.01 μM | Typical value for Michaelis-Menten (MM) parameter for phosphorylation is 0.01 μM [4] |
| *k_AD_* | 72 μM / hr | Typical value dephosphorylation rate constant assuming a constant phosphatase concentration is 720 μM/hr [4] |
| *K_AD_* | 0.01 μM | Typical value for MM parameter for dephosphorylation [4] |
| *k_GP_* | 360 / hr | Typical value phosphorylation rate constant is 3600/hr [4] |
| *K_GP_* | 0.01 μM | Typical value for MM parameter for phosphorylation [4] |
| *k_GD_* | 72 μM / hr | Typical value dephosphorylation rate constant assuming a constant phosphatase concentration is 720 μM/hr [4] |
| *K_GD_* | 0.01 μM | Typical value for MM parameter for dephosphorylation [4] |
| *k_MS_^*^* | 2.3 /hr | These values are adjusted together so that   1. The peak level is at least 3 times higher than the basal level. 2. The level of the second hump is about 60 % of the peak level   [1] |
| *K_MS_* | 0.01 μM / hr |
| *k_MT_* | 0.4 /hr |
| *K_MT_* | 0.01 μM / hr |
| *d_M_* | 2.08 / hr | [5,6] |
| *d_MS_* | 0.35 / hr | Myc’s half-life is increased by 6fold upon phosphorylation at Ser62[5,6] |
| *d_MT_* | 2.08 / hr | Assumed to be the same as *d_M_* |

Initial Akt = 0.6 μM and initial Gsk3β = 0.6 μM.

*Erk_R_*: Residual Erk level. Adjusted to 10 percent of the maximal Erk level. To reflect experimental observations in our model, residual Erk level is required. Without Erk_R_, our model cannot generate the second peak of Myc activity.

*PI3K_R_*: Residual PI3K level. Adjusted to 10 percent of the maximal PI3K level.

^*^ *Erk_Max_* and *k_MS_* together represent Erk’s phosphorylation efficiency of Myc at Ser62. If *k_MS_*[*Erk_Max_*] is sufficiently large, our model can generate the initial peak of Myc activity as observed in [1]. Further increase in *k_MS_*[*Erk_Max_*] does not significantly change the initial peak of Myc activity, suggesting that Erk’s phosphorylation efficiency is operating at saturation.

^**^ Prevention of Myc destabilization by Gsk3β depends on PI3K’s ability to inhibit Gsk3β activity. A sufficiently strong PI3K pulse is needed to prevent Myc from destabilization and to generate the second round of the Myc accumulation.

**References:**

1. O'Donnell KA, Wentzel EA, Zeller KI, Dang CV, Mendell JT (2005) c-Myc-regulated microRNAs modulate E2F1 expression. Nature 435: 839-843.

2. Sasagawa S, Ozaki Y, Fujita K, Kuroda S (2005) Prediction and validation of the distinct dynamics of transient and sustained ERK activation. Nature Cell Biology 7: 365-U331.

3. Jones SM, Klinghoffer R, Prestwich GD, Toker A, Kazlauskas A (1999) PDGF induces an early and a late wave of PI 3-kinase activity, and only the late wave is required for progression through G1. Current Biology 9: 512-521.

4. Kholodenko BN (2006) Cell-signalling dynamics in time and space. Nature Reviews Molecular Cell Biology 7: 165-176.

5. Sears R, Nuckolls F, Haura E, Taya Y, Tamai K, et al. (2000) Multiple Ras-dependent phosphorylation pathways regulate Myc protein stability. Genes & Development 14: 2501-2514.

6. Lepique AP, Moraes MS, Rocha KM, Eichler CB, Hajj GNM, et al. (2004) c-Myc protein is stabilized by fibroblast growth factor 2 and destabilized by ACTH to control cell cycle in mouse Y1 adrenocortical cells. J Mol Endocrinol 33: 623-638.
