# Supplementary material for: Sensing and Integration of Erk and PI3K Signals by Myc
Source: PLoS Comput Biol. 2008 Feb 29;4(2):e1000013. doi: 10.1371/journal.pcbi.1000013 (PMC2265471; doi:10.1371/journal.pcbi.1000013)
Supplement: Table S7 — Parametric Sensitivity without ultrasensitivity (0.03 MB DOC) [file pcbi.1000013.s012.doc]

Table S7: Parametric Sensitivity without ultrasensitivity^*^

| **Parameters** | **Sensitivity =** $\log_{10}\left(\frac{Potency_{Increase}}{Potency_{Decrease}}\right)$ |
| --- | --- |
| Myc synthesis rate constant (*k_M_*) | 4.6 |
| Myc degradation rate constant (*d_M_*) | -1.3 |
| Rate constant for Myc phosphorylation at Ser62 (*k_MS_*) | 0.81 |
| Myc^Ser62^ degradation rate constant (*d_MS_*) | -0.79 |
| Rate constant for Myc phosphorylation at Thr58 (*k_MT_*) | -0.64 |
| Myc^Thr58^ degradation rate constant (*d_MT_*) | -0.61 |
| Gsk3β phosphorylation rate constant (*k_GP_*) | 0.56 |
| Gsk3β dephosphorylation rate constant (*k_GD_*) | -0.56 |
| Akt dephosphorylation rate constant (*k_AD_*) | -0.41 |
| Akt phosphorylation rate constant (*k_AP_*) | 0.41 |
| MM constant for Myc^Ser62^ phosphorylation (*K_MS_*) | -0.12 |
| MM constant for Myc^Thr58^ phosphorylation (*K_MT_*) | 0.10 |

*Sensitivity to other parameters was equal to or smaller than 0.1. These include Michaelis-Menten constants for Myc^Thr58^ phosphorylation (*K_MT_*), Gsk3β phosphorylation (*K_GP_*) / dephosphorylation (*K_GD_*), and Akt phosphorylation (*K_AP_*) / dephosphorylation (*K_AD_*).
