# Supplementary material for: Sensing and Integration of Erk and PI3K Signals by Myc
Source: PLoS Comput Biol. 2008 Feb 29;4(2):e1000013. doi: 10.1371/journal.pcbi.1000013 (PMC2265471; doi:10.1371/journal.pcbi.1000013)
Supplement: Text S1 — (0.05 MB DOC) [file pcbi.1000013.s013.doc]

**Supplementary Material for**

**Sensing and integration of Erk and PI3K signals by Myc**

Tae Lee, Guang Yao, Joseph Nevins, and Lingchong You

*1. Experimental observations of Erk, PI3K, and Myc signaling patterns*

Temporal behaviors of Erk (Table S1), PI3K (Table S2) and Myc (Table S3) signaling patterns are variable with cell lines and growth conditions. Nonetheless, these signals share common features in different conditions. For example, Erk exhibits an early, transient peak followed by variable residual level. PI3K also exhibits an early, transient peak, but has a second peak after some time delay. Myc reaches its initial peak approximately 2 hours after induction, and is either sustained or elevated depending on the input signal patterns

*2. Model development and assumptions*

We have developed a mathematical model to account for the key interactions outlined in Figure S1. Our model consists of a set of ordinary differential equations (ODEs) which are constructed from the reaction kinetics in Table S4 and parameters in Table S5. The ODEs are implemented and simulated in Dynetica [1] and Matlab. To best reflect experimental observations, we estimate some parameters from related or similar kinetic processes. Others are adjusted so that our model predictions will agree with existing biological data.

Due to lack of detailed quantitative analysis of the PI3K pathway, many rate parameters for our modeled reactions are unavailable. To address this limitation, we have adopted base parameter values that fall into the range of values typically used for other phosphorylation or dephosphorylation reactions (Table S5). In adopting these parameter values for our system, we made an implicit assumption that different types of phosphorylation-dephosphorylation (Ph-dePh) cycles share similarity, not only in terms of the mechanism, but also in terms of parameter values. It is possible that the adopted base parameter values may differ significantly from the true values. To address this caveat, we carried out extensive sensitivity analysis to test the impact of the uncertainty in these parameters. Our results suggest that overall, our conclusions will hold despite significant changes in these parameters (up to 100fold change for each parameter, while holding the others constant). Further tests of the modeling predictions against experiments will help constraining these parameters.

We have used Michaelis-Menten kinetics to model phosphorylation or dephosphorylation reactions, which is a well-established practice in modeling signaling pathways [2-5]. Under certain circumstances, the Michaelis-Menten kinetics may deviate from true dynamics. Overall, our model predictions on the response of Myc to varying input signals (Erk and PI3K) have been qualitatively consistent with existing experimental observations. Due to the lack of quantification of signaling dynamics leading to Myc activity (especially at the individual reaction level), it is unclear how, at the quantitative level, the Michaelis-Menten kinetics may faithfully reflect the underlying kinetics. If our model’s predictions deviate significantly from more quantitative experimental observations, it may be necessary to formulate the model using alternative methods, such as the “total quasi-steady state assumption” (tQSSA) [6].

*3. Parametric senstivity analysis*

To quantify the change in Myc accumulation due to parameteric changes, we use Myc potency, the area under the Myc temporal profile. Sensitivity is the amount of change in Myc potency to a 100fold change in each parameter. To be more specific, we calculated Myc potency for 10fold increase and decrease in each parameter. Then we took the logarithmic ratio between the largest Myc potency and the smallest Myc potency. Therefore, the positive values in Table S6 indicate logarithmic fold-increase in Myc potency over 100fold parameter change. The negative values indicate logarithmic fold decrease. Sensitivity of ‘0’ represents no change, and ‘1’ represents 10 fold changes in Myc potency.

Myc potency had a “gradient” of sensitivity to various parameters. Many parameters had little impact on Myc potency when the input Erk and PI3K signals were strong. For a 100fold change in each of these, the corresponding change in the potency was less than 10fold. Many of these are involved in the PI3K signaling cascade. These include the Michaelis-Menten (MM) constants for Akt and Gsk3β phosphorylation and dephosphorylation. The insensitivity of Myc potency to these parameters is probably because each stage operated with zero-order ultrasensitivity for the base parameter setting [7]. That is, each phosphorylation-dephosphorylation cycle acts as a digital switch in response to changes in kinase and phosphatase concentrations (Figure S2). This switching behavior occurs under the following conditions: 1) the phosphorylation and dephosphorylation rates are close to each other, and 2) the protein concentration is much greater than the MM constants. As long as parameter changes did not cause loss of ultrasensitivity, the switching behavior of each stage was not significantly changed (Figure S2).

To investigate ultrasensitivity in the PI3K pathway further, we evaluate contribution of each phosphorylation-dephosphorylation (Ph-dePh) cycle of the cascade towards overall ultrasensitivity. In particular, we characterize parameter dependence of ultrasensitivity in each Ph-dePh cycle, and how this affects the cascade’s overall input/output response. We first start with our base model, where we assume ultrasensitivity in the Akt (*K_AD_*=*K_AP_* = *K_A_*=0.01) and Gsk3β (*K_GD_*=*K_GP_*=*K_G_*=0.01) Ph-dePh steps (blue line in Figure S5A,B). Consequently, the overall cascade response is ultrasensitive. When the ultrasensitivity in Akt Ph-dePh step is lost (*K_A_*=1, red line in Figure S5A), the overall cascade response is not significantly affected as long as Gsk3β Ph-dePh cycle (*K_G_*=0.01) remains ultrasensitive (Figure S5B). When the ultrasensitivity in Gsk3β Ph-dePh cycle is lost (*K_G_*=1, Figure S5C), the overall ultrasensitive response can be maintained if Akt Ph-dePh cycle is ultrasensitive (blue line in Figure S5C). Otherwise, the overall system response loses ultrasensitivity (red line in Figure S5C). Here we limit our analysis to two extreme cases where ultrasensitivity is present or absent.

We note that overall input/output response is a convolution of the input/output responses between PI3K🡪Akt and Akt🡪Gsk3β. Ultrasensitivity may arise if the ‘gating’ between these responses matches. In this case, it has been shown that even without ultrasensitivity in each level of the cascade, ultrasensitivity may arise [8].

Although the ultrasensitivity in the PI3K pathway may facilitate noise-resistance, it is not absolutely required for insensitivity to parameter changes. The noise-resistant feature of the model also arises from the inputs operating at the saturation level. If the input level is sufficiently large, minor increase or decrease in the input level may not have much impact on the output response. This is clear Table S7, where we repeat sensitivity analysis assuming absence of ultrasensitivity in Akt (*K_A_*=1) and Gsk3β *(K_G_*=1) Ph-dePh cycles. The rate constants for Myc phosphorylation at Ser62 (*k_MS_=*3.4/hr) and Thr58 (*k_MT_=*1.08/hr) are adjusted to match Myc’s steady-state level with our base model. Although a minor increase is observed in some parameters (*k_MT_*, *d_MT_*, *k_GP_*, and *k_GD_*) the results in Table S7 do not significantly deviate from those in Table S6 as long as the inputs are sufficiently strong. For example, Myc potency is sensitive to parameters directly involved in Myc protein modification or degradation. However, many parameters involved in the PI3K signaling cascade have little impact on Myc potency. In contrast, when the input signals are weak, the sensitivity is overall increased. Ultrasensitivity with increasing effective Hill coefficient may ‘shrink’ the sensitive region in input/output response curve, allowing wider range of input values for noise-resistance. However, Michaelis-Menten response is sufficient to confer resistance to minor fluctuations when the input value is large. This aspect is also evident in the analysis of the simplified dual-kinase module, which assumes no cooperativity in phosphorylation of Myc isoforms.

**References**

1. You LC, Hoonlor A, Yin J (2003) Modeling biological systems using Dynetica - a simulator of dynamic networks. Bioinformatics 19: 435-436.

2. Kholodenko BN (2006) Cell-signalling dynamics in time and space. Nature Reviews Molecular Cell Biology 7: 165-176.

3. Kholodenko BN (2000) Negative feedback and ultrasensitivity can bring about oscillations in the mitogen-activated protein kinase cascades. European Journal of Biochemistry 267: 1583-1588.

4. Igoshin OA, Goldbeter A, Kaiser D, Oster G (2004) A biochemical oscillator explains several aspects of Myxococcus xanthus behavior during development. Proceedings of the National Academy of Sciences 101: 15760-15765.

5. Hatakeyama M, Kimura S, Naka T, Kawasaki T, Yumoto N, et al. (2003) A computational model on the modulation of mitogen-activated protein kinase (MAPK) and Akt pathways in heregulin-induced ErbB signalling. Biochem J 373: 451-463.

6. Ciliberto A, Capuani F, Tyson JJ (2007) Modeling Networks of Coupled Enzymatic Reactions Using the Total Quasi-Steady State Approximation. PLoS Computational Biology 3: e45.

7. Goldbeter A, Koshland DE, Jr. (1981) An amplified sensitivity arising from covalent modification in biological systems. Proceedings of the National Academy of Sciences of the United States of America 78: 6840-6844.

8. Huang C-YF, Ferrell JE, Jr. (1996) Ultrasensitivity in the mitogen-activated protein kinase cascade. PNAS 93: 10078-10083.
